# Detection of *Francisellaceae* and the differentiation of main European *F. tularensis* ssp. *holarctica* strains (Clades) by new designed qPCR assays

**DOI:** 10.1186/s12866-025-03751-9

**Published:** 2025-01-17

**Authors:** Kristin Köppen, Kerstin Rydzewski, Julia Zajac, Marwah Al-Senwi, Sema Evcimen, Darius Schulze, Daniela Jacob, Klaus Heuner

**Affiliations:** 1 Cellular Interactions of Bacterial Pathogens, Centre for Biological Threats and Special Pathogens, Highly Pathogenic Microorganisms (ZBS 2), Robert Koch Institute, Seestraße 10, 13353 Berlin, Germany; 2 National Consultant Laboratory for Francisella Tularensis, Centre for Biological Threats and Special Pathogens, Highly Pathogenic Microorganisms (ZBS 2), Robert Koch Institute, Berlin, Germany

**Keywords:** Tularemia, *Francisellaceae*, *Francisella tularensis* ssp*. holarctica*, qPCR, Diagnostic

## Abstract

**Background:**

The zoonotic and highly infectious pathogen *Francisella tularensis* is the etiological agent of tularemia. Tularemia in humans is mainly caused by *F. tularensis* subspecies *tularensis* and *holarctica*, but *Francisella* species like *F. novicida, F. philomiragia, F. hispaniensis* and others are known to cause tularemia-like infections in immunocompromised humans. In addition to these *Francisella* species, further genera of the family *Francisellaceae* have been described, such as *Allofrancisella, Parafrancisella* and *Pseudofrancisella*, but less is known about the distribution and putative virulence of these genera. The methods currently available were not made for a fast and easy detection of all these strains and genera of *Francisellaceae*.

**Results:**

We developed a multiplex quantitative real-time PCR assay that can accurately detect all genera of *Francisellaceae*, including *Francisella*, *Francisella*-like endosymbionts, *Allofrancisella, Parafrancisella* and *Pseudofrancisella.* In addition, we developed a qPCR assay to differentiate the major clades (B.4, B.6 and B.12 [B.71 and B.72]) of *F. tularensis* ssp. *holarctica* strains. Both primer sets were shown to work on isolated DNA out of human and tick samples.

**Conclusion:**

Since the developed qPCRs are able to detect all genera of *Francisellaceae* tested, an easy and fast identification of opportunistic *Francisella* strains causing tularemia-like symptoms in humans or animals is possible now. The application of these qPCR assays will thus improve the capability for clinical diagnostics and molecular typing during epidemiological investigations.

**Supplementary Information:**

The online version contains supplementary material available at 10.1186/s12866-025-03751-9.

## Background

**Fig. 1:**
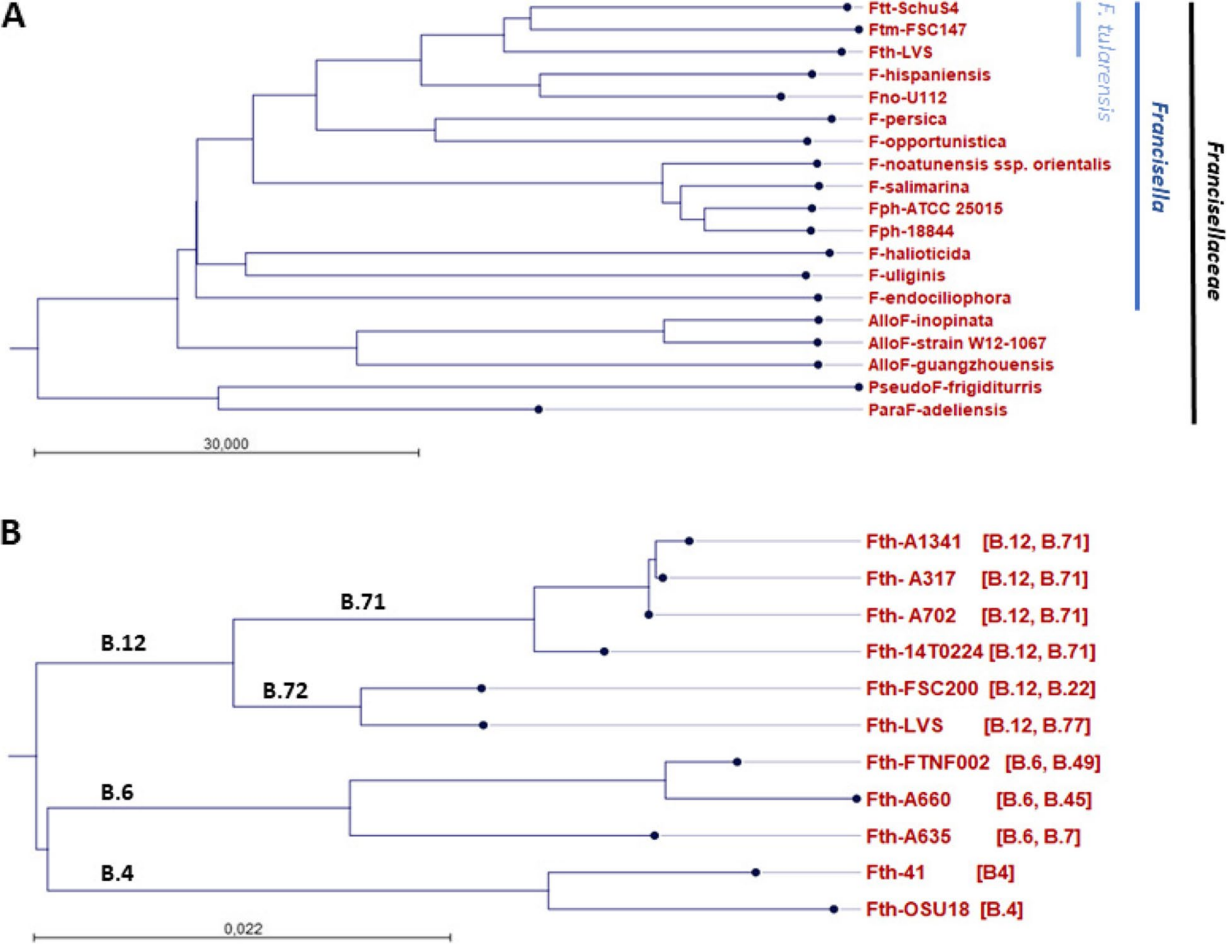
Whole genome alignment neighbor-joining tree of *Francisellaceae*. **A** Representative genomes of *Francisellaceae* strains: *F. tularensis* ssp. *mediasiatica* (Ftm-FSC147, CP000915), *F. tularensis* ssp. *tularensis* (AJ749949.2), *F. tularensis* ssp. *holarctica* (LVS, NC_0078801), *F. novicida* (Fno-U112, NC_008601), *F. hispaniensis* (CP018093), *F. opportunistica* (CP022377), *F. persica* (CP013022), *F. philomiragia* (Fph 18844, CP063138; ATCC25015, CP010019), *F. noatunensis* ssp. *orientales* (CP003402), *F. salimarina* (CP076680), *F. endociliophora* (CP009574), *F. halioticida* (CP022132), *F. uliginis* (CP016796), *Allofrancisella* (AlloF) *guangzhouensis* (CP010427), *A. inopinata* (CP038241), *Allofrancisella* sp. W12-1067 (AWHF01000000), *Pseudofrancisella* (PseudoF) *frigiditurris* (CP009654) and *Parafrancisella* (ParaF) *adeliensis* (FSC1325, CP043424) were used for the alignment. **B** Genomes of *Fth* strains belonging to *Fth* subclade B.4 (Fth-41 [25]); OSU18, CP0000000), B.6 (FTNF002, CP000803; *Fth*-A-660, [25]), B.12, B.71 (A-1341, CP098826; A-317, A-702, [25]), B.12, B.72 (LVS, NC_0078801; OSU18, CP000437)

*Francisella tularensis* is the causative agent of the zoonotic disease tularemia. *F. tularensis* has been found in more than 200 animal species, including rabbits, dogs and ticks. Transmission to humans can occur by handling contaminated animals, food or water but also by insect/arthropod bits (mosquitos and ticks). Clinical manifestations range from flu-like symptoms to severe pneumonia and depend on pathogen’s entry [1–5], resulting in the following forms: ulceroglandular or glandular (most common), oropharyngeal, ocularglandular and respiratory, as well as the rare thypoidal form [6]. *F. tularensis* belongs to the family *Francisellaceae* which consists of four valid genera*: Francisella, Parafrancisella, Allofrancisella*, and *Pseudofrancisella* [7–9] (Fig. 1A), and further candidates as *Nebulibacter, Cyteiniphilum, Caedibacter*, and *Fangia* [7, 10, 11]. The genus *Francisella* comprises more than 20 species, including environmental species (like *F. endociliophora* [12], *F. salimarina* [13]), fish pathogens *(F. noatunensis, F. philomiragia* [14]), *Francisella*-like endosymbionts (FLEs, [3, 15]) as well as opportunistic species like *F. opportunistica* [16], *F. novicida* [17], *F. salimarina* [18]) and *F. hispaniensis* [19]) which can also induce tularemia-like symptoms in immunosuppressed humans. The other three genera consist of several environmental strains (for example *Allofrancisella* sp. strain W12-1067 [20]. *Francisellaceae* species are predominantly distributed on the northern hemisphere, but *Francisella* species has been yet found also in Australia and Africa [19, 21]. The causative agent of tularemia, *F. tularensis*, comprises three subspecies: *F. tularensis* ssp. *tularensis* (*Ftt*), *F. tularensis* ssp. *holarctica* (*Fth*) and *F. tularensis* ssp. *mediasiatica* (*Ftm*) which differ in their geographic distribution and virulence. The highly virulent *Ftt* is found exclusively in North America; *Ftm* is less virulent and distributed in the central Asian region (Kazakhstan, Uzbekistan) and Russia [22–24]. In Europe, only *Fth* has been found to cause tularemia in human and animals, but is also present on the whole northern hemisphere [1, 2].

As mentioned above, infections with opportunistic *Francisella* species occur also in Europe. Recently, a child, suffering from chronic granulomatous disease, has been infected by *F. philomiragia* while surfing in the Danish North Sea [26] and a diabetic man with myeloproliferative disorders has also been infected by *F. philomiragia* probably through saltwater from the French North Sea [27]. One study reported that in France 19% of the ticks (*Dermacentor marginatus*) investigated were positive for *F. philomiragia* using a PCR-based assay [28] and a further study detected *F. philomiragia* in coastal waters in Norway [29]. The mentioned studies indicate that opportunistic species of *Francisellaceae* are present in Europe and the number of infections by opportunistic *Francisella* species may be underestimated.

**Fig. 2:**
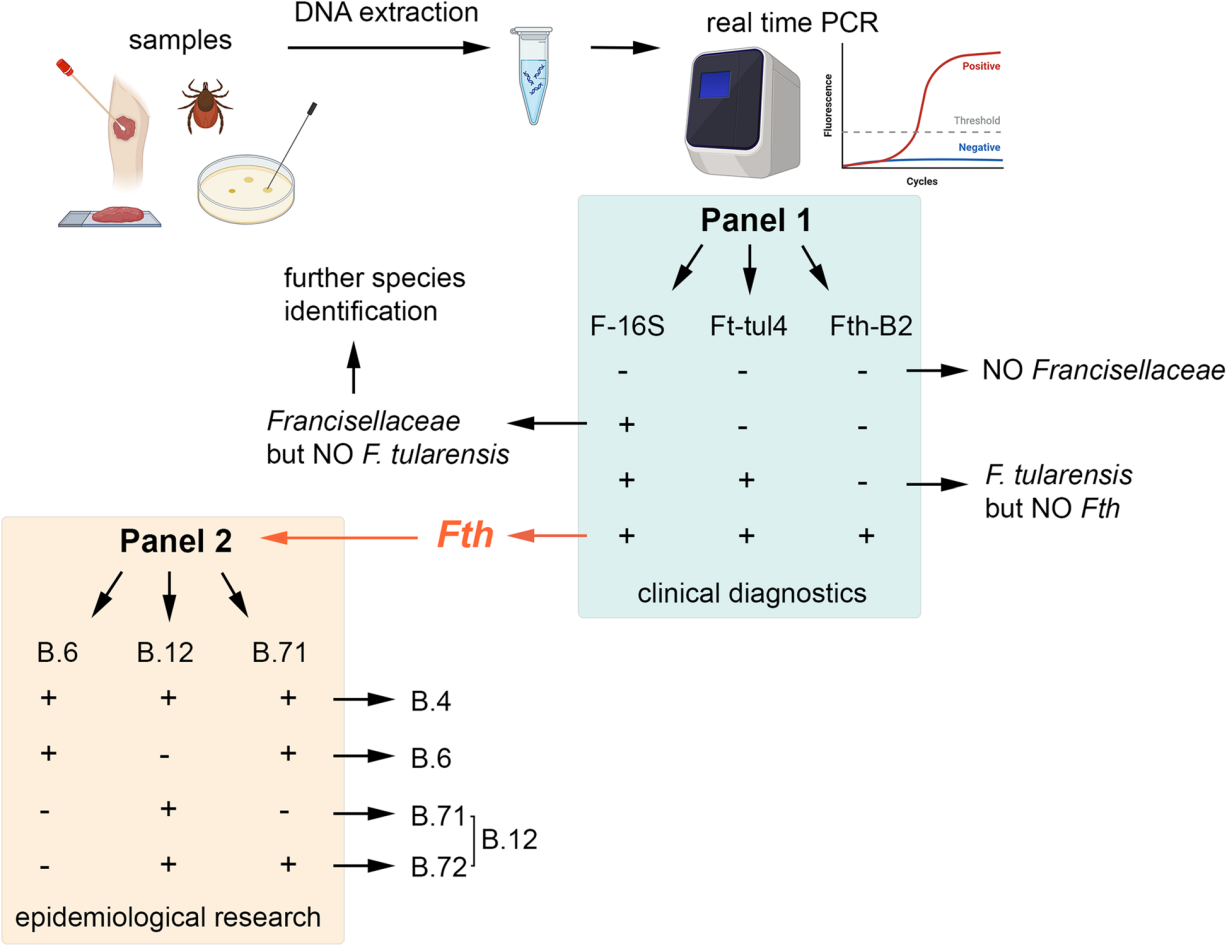
Workflow of PCR panel applications. Clinical specimens (like bacterial isolates, lymph nodes, wound swabs, paraffin-fixed tissues) and environmental samples (ticks) are used to extract DNA. The isolated DNA is tested for *Francisellaceae* DNA using PCR Panel 1 with F-16S, Ft-tul4 and Fth-B2 as part of the clinical diagnostics (green background). If the samples are *Fth* positive (all three sets show amplification), panel 2 can be used to discriminate the main *Fth* clades in the context of epidemiological research purposes (orange background). + : positive qPCR result with Ct-values below 40; -: negative PCR result without Ct-value. (Figure created in biorender.com)

Generally, the diagnosis of tularemia is challenging and often delayed, because tularemia is a rare disease in humans leading to a low awareness for this disease. Molecular methods (such as quantitative real-time polymerase chain reaction (qPCR) assay, sequencing and matrix-assisted laser desorption/ionisation time-of-flight mass spectrometry (MALDI)) and serological tests (like enzyme-linked immunosorbent assay and immunoblots) are commonly used to diagnose tularemia [30, 31]. For the identification of the subspecies of *F. tularensis*, different PCR-based methods are used including the conventional RD1 PCR [32] and multiplex qPCR assays as described by Gunnell et al. 2012 and Larson et al. 2020 for instance [33, 34]. The RD1 assay targets the “Region of Differentiation 1” resulting in amplicons of different sizes for the different subspecies [32]. However, uncommon *Francisellaceae* species causing infections with tularemia-like symptoms may be insufficiently diagnosed, since they are not detected by standard qPCR assays, which mostly detect only *F. tularensis* and *F. novicida* [34–38]. For example, both *F. philomiragia* infections mentioned above were identified by MALDI-Tof or 16S and 23S rDNA sequencing of bacterial isolates [26, 27]. For research use, different methods were published detecting at least some other *Francisella* species [39–41], but not all members of the family of *Francisellaceae*. Therefore, we aimed to extend the research and diagnostic profile detecting all species of the family of *Francisellaceae* by qPCR (see Fig. 2, panel 1).

In Europe, *Fth* isolates are grouped into Biovar I and II according to their erythromycin resistance. Based on phylogenetic and canonical SNP analysis, isolates can be divided into three basal clades: B.4, B.6 (representing biovar I) and B.12 (representing biovar II, see Fig. 1B) [42–44]. These basal clades are found in Germany. However, isolates belonging to B.4 are rarely found, B.6 strains are more common in northeastern parts and B.12 strains in southwestern parts of Germany [25, 45]. Basal clade B.12 can be further separated into B.71 and B.72 [46–48]. Recently, we showed that isolates of B.6, B.71 and B.72 possess specific growth pattern in liquid media and that B.6, B.71 and B.72 can be discriminated by their proteome, in addition to their genome [48]. Also, it is assumed that B.6 and B.12 clades may display differences in pathogenicity [25, 49–52]. So far, mainly bacterial isolates are used for melt-mismatch amplification mutation assay (SNP based) or whole genome sequencing (WGS) and subsequent phylogenetic analysis for grouping into clades [40, 44, 53]. However, there is a discrepancy of tularemia cases per year and the number of obtained human *Fth* isolates. The discrepancy can be attributed to two primary factors: firstly, a significant proportion of tularemia cases are confirmed solely on the basis of immunological tests (serological diagnosis; [30, 31]), and secondly, the diagnosis of tularemia is frequently delayed, resulting in patients being treated with antibiotics prior to the confirmation of the infection. It is reported, that *F. tularensis* isolates are obtained in less than 10% of the patients [54–59]. To include as many tularemia cases as possible in the phylogenetic and epidemiological analysis, we developed a qPCR discriminating between B.4, B.6, B.71 and B.72 strains to differentiate *Fth* isolates for the main basal phylogenetic clades detected in Germany (Fig. 2, panel 2). This panel can be used although no isolate was obtained from the patient or probe.

## Methods

### Specimens and DNA preparation

*Francisellaceae* strains used in this study, listed in Table S1, were cultivated on medium T agar plates supplemented with coal and hemoglobin (MTKH agar plates) [60]. *Fth* isolates (see Table 2) were obtained from human specimen in our National Consultant Laboratory for *Francisella tularensis* in human medicine (ZBS 2, Robert Koch Institute, Berlin). *Pseudofrancisella* strains were provided by the State Office for Nature, Environment and Consumer Protection North Rhine-Westphalia (LANUV, Recklinghausen, Germany). In the course of the routine diagnostic for tularemia, different clinical samples were obtained from patients (see Fig. 2) and ticks were used to evaluate the different qPCR panels. Genomic DNA was isolated from bacterial cultures and human material using DNeasy Blood & Tissue Kit (Qiagen, Hilden, German) according to manufacturer’s instruction. An internal control DNA (10 µl of 10^3^ genome equivalent (GE) KoMa [61]) was added to the human samples prior to the lysis step. DNA from non-*Francisellaceae* species were provided by ZBS2 and are listed in Table S2. DNA from ticks were isolated using the blackPREP Tick DNA/RNA Kit (IST Innuscreen GmbH, Germany) and the SpeedMill homogenizer (Analytik Jena AG, Germany) according to manufacturer's instructions.

### Primer and probe design

- 1. *Francisellaceae*. To be able to detect all members of the family *Francisellaceae* (see above), we selected the 16S rRNA gene and the 30S ribosomal protein gene (rpsL) as possible templates for primer design. Two different primer/probe sets for the 16S rRNA gene (F-16S-1 and F-16S-2; see Table 1 and Table S3) as well as one set amplifying the rpsL gene (F-30S) were generated using a general Blast search strategy searching for specific binding within all the genomes of interest and to avoid cross-annealing of the primer/probe sequences with DNA of non-*Francisellaceae* species. In silico, all three sets were found to be suitable for PCR analysis and therefore they were tested first in a conventional PCR and then in qPCR.
- 2. *Fth* clades. Nucleotide sequences of draft genomes of 78 *Fth* strains (see [45]) (plus genomes of OSU18 and FTNFOO2) including 45 B.6 strains, 32 B.12 strains (4 B.71 and 28 B.72) and one B.4 strain were aligned using Geneious Prime (Mauve Alignement) and analysed for In/Del regions by manual inspection [48]. Such regions were used to generate primer sequences with a melting temperature (Tm) of about 57–62 °C. Sequences were screened by Blast search for single binding within the genome and for less binding to other strains or species. Two B.6-specific targets (FTL_0701 and FTL_1504) and four B.12-specific targets (FTL_0742, _0734, _1896, _1896) were selected. Probe sequences of about 30–35 bp with a Tm of about 10 °C higher than Tm of the respective primers were generated and also in silico evaluated by Blast search (Table 1 and Table S3).

**Table 1:** Primer and probe sequences for qPCR assays

PCR assay	Name	Primer / probe	Sequence and labeling (5´- > 3´)	Target	Description	Reference
Ft-tul4	Ft-tul4-F	forward primer	agattacaatggcaggctcc	tul4	detection of *F. tularensis* and *F. novicida*	[46]
	Ft-tul4-R	reverse primer	agctgtccacttaccgctaca			
	Ft-tul4-P	probe	Cy5- ttctaagtgccatgatacaagcttcccaa-BHQ-2			
Fth-B2	Fth-B2-F	forward primer	cctatccaatactccgagttagt	FTS_0806	*Fth*-specific	[34]
	Fth-B2-R	reverse primer	aaatcaaaagaagagttaaaacaagc			
	Fth-B2-P	probe	FAM-ctctggccagttatttttatcaaagccag-BHQ-1			
F-16S	F-16S-F2	forward primer	tgacaggtgctgcacggctgt	16S	*Francisellaceae*-specific	this study
	F-16S-R2	reverse primer	gcagccctctgtaatacccatt			this study
	F-16S-P2	probe	Cy5.5-acccaacttaatgatggtaactatcaatag-BHQ-3			this study
B.6	B.6-E–F	forward primer	atgagtaattcattattctcctagattgaaa	FTL-1504	discrimination of *Fth* B.4, B.6 and B.12 clades: B.12-C-P (FAM) signal = B.12 strain B.6-ES-P (Cy5) signal = B.6 strain B.12-C-P (FAM) and B.6-ES-P (Cy5) signal = B.4 strain	this study
	B.6-E-R	reverse primer	gtgataaataaaaatctatagcataacatcc			this study
	B.6-ES-P	probe	Cy5- aatgattgataatagaacctacccctataccaact-BHQ-2			this study
B.12	B.12-C2-F	forward primer	ggtgcttgagtcataggtggc	FTL-1896		this study
	B.12-C2-R	reverse primer	taacaagtgtaggtgcagcaa			this study
	B.12-C-P	probe	FAM- tcgccatattgaatattagttccagtagctgttag -BHQ-1			this study
B.71	B.71-F2	forward primer	ttaaaaattgcaagtccatgtcta	Intergenic region, deletion, 888,400^a^	*Fth* clade B.71 specific (no signal for B.71)	this study
	B.71-R2	reverse primer	caacacctaaagcttctaatgatttcacataatgactt			this study
	B.71-P2	probe	Hex-taatattcctcaacagttaactatatctagcaaaacaatta-BHQ-1			this study
KoMa	KoMa2-F	forward primer	ggtgatgccgcattattactagg		internal amplification control	[61]
	KoMa2-R	reverse primer	ggtattagcagtcgcaggctt			
	KoMa2-P	probe	Joe-ttcttgcttgaggatctgtcgtggatcg-BHQ-1			

^a^ Nucleotide position according to the genome of *Fth* A-1341 [48]

We further generated two primer/probe pairs (B.71-F1/R1/P1 and B.71-F2/R2/P2) which are negative for an amplification product using DNA of B.71 strains (Table 1 and Table S3).

All primer pairs were tested in a qPCR for functionality, see below. Sequences of all primers and probes are given in Table 1 and Table S3.

### Conventional PCR

Conventional PCR was carried out using a Thermocycler TRIO-Thermoblock (Biometra, Göttingen, Germany) and the Q5® High-Fidelity 2X Master Mix (New England Biolabs, Massachusetts, US) according to manufacturer's specifications. In general, initial denaturation was performed at 98 °C for 30 s and final extension was performed at 72 °C for 2 min. The cycling conditions (35 cycles) were 98 °C for 10 s, 60 °C for 30 s and 72 °C for 30 s/kb, and ∼ 100 ng of template DNA was used. Oligonucleotides were obtained from Eurofins MWG Operon (Germany).

### Quantitative real-time PCR

Quantitative real-time PCR assays (qPCR, TaqMan technology) were performed with oligonucleotides and probes which are listed in Table 1 and Table S3. qPCRs were run in a total volume of 25 µl comprising 5 µl DNA, 6.25 µl TaqMan Environmental MasterMix 2.0 (ThermoFisher, Germany), 10 µmol/µl primers (0.75 µl each) and 10 µmol/µl probes (0.25 µl each). In each qPCR assay the internal control (KoMa) was added either during the DNA isolation (see above) or during the qPCR-mix preparation (5 µl of 10^3^ GE KoMa per amplification). Primer were obtained from Eurofins MWG Operon (Germany) and probes from Metabion (Germany). KoMa plasmid DNA was provided by ZBS 2. Samples were analysed in technical duplicates per run. Amplification was performed at Bio-Rad CFX96 cycler (Bio-Rad Laboratories, Germany) with following instructions: initial denaturation step at 95 °C for 10 min, followed by 40 cycles containing a denaturation step at 95 °C for 15 s and a combined prime annealing and elongation step at 60 °C for 60 s.

## Results and discussion

### Implementation of qPCR detecting all strains of* Francisellaceae*

Due to the increasing number of environmental and opportunistic *Francisella* strains in the last years, it is highly important to identify all representatives of the family *Francisellaceae* in clinical and environmental samples. Therefore, three different primer/probe combinations were designed detecting all members of the family *Francisellaceae* investigated, as described above. The primer/probe sets targeting the 16S and the 30S DNA sequence and were initially tested using conventional PCR. All three sets were found to be specific and were subsequently evaluated in qPCR experiments using a small set of *Francisellaceae* strains of different genera (data not shown). The primer/probe combination F-16S-F2/R2/P2 was found to be the most promising specific candidate due to highest sensitivity and efficiency. Selected strains (*Fth* A-271, A-660, A-271) were used to determine primer and probe concentrations in the qPCR resulting in the concentration of 10 µmol/µl per primer and probe (for more information see Method section). The F-16S-F2/R2/P2 set, named F-16S, was further tested using in total 45 different *Francisellaceae* strains of the four genera *Allofrancisella*, *Pseudofrancisella, Parafrancisella* and *Francisella*. Furthermore, a selection of 26 bacterial non-*Francisellaceae* environmental, pathogenic and opportunistic strains was tested (see Table S2). The results demonstrated that all tested *Francisella* species as well as strains of *Allofrancisella*, *Pseudofrancisella,* and *Parafrancisella* were successfully identified at high (5 × 10^6^ genome equivalents [GE]) and low (5 × 10^2^ GE) DNA concentrations in the qPCR. In contrast, all tested non-*Francisellaceae* strains showed no amplification in the qPCR (at high and low DNA concentration). The results demonstrated that this F-16S qPCR set can be used for further evaluation experiments using diagnostic and environmental (tick) samples (see below).

### Implementation of a qPCR discriminating basal clade B.4, B.6 and B.12 (B.71 and B.72) of Fth strains

*F. tularensis* is the causative agent of tularemia. In Germany yet only *F. tularensis subspecies holarctica* was identified in tularemia cases in humans and wild animals. This subspecies consists of three basal clades (B.4, B.6 and B.12, Fig. 1B), which differ in their geographical distribution and likely in their virulence [44, 45, 49–52]. Therefore, an easy and fast discrimination between these clades might be beneficial. The here described PCR-based subtyping would facilitate surveillance and epidemiological analysis as well as assist in understanding the putative association between specific clades and clinical symptoms, as well as the geographic distribution of clades in Germany. To achieve this, eight target sequences were chosen with deletions either in B.6 (FTL_1896, _0742, _0734, _1896), in B.72 (FTL_1504, _0701) or B.71 strains (CP098826.1 NT position 888,444; [48]). Primers and probes were designed as described above. All combinations were individually tested in conventional PCR and qPCR with a small set of *Francisellaceae* strains (data not shown). The most promising set for each primer/probe combination was selected (B.6-E–F/R/ES-P named B.6, B.12-C2-F/R/P named B.12, and B.71-F2/R2/P2 named B.71, see Table 1) due to highest sensitivity and efficiency; and further tested in more detail using high and low-concentrated DNA (10^2^ – 10^6^) of 45 *Francisellaceae* strains (see above) and 26 non-*Francisellaceae* strains (Table 2). The qPCRs were performed in single as well as in multiplex-assays (see methods, qPCR). As expected, the new primers and probes did not show an amplification curve for any of the non-*Francisellaceae* strains at high and low DNA concentration, but some of them showed a positive result for *Ftm, Ftt* and *F. novicida* (Table 2), which can be neglectable, since these primers are only designed for the subtyping of *Fth* positive-tested strains and samples. Importantly, the PCR panel 2 is not recommended to be used for other *Francisella* strains. The B.6 set successfully detected all tested B.4 and B.6 *Fth* strains and the B.12 set successfully detected B.4 and B.12 (B.71 and B.72) strains (Table 2). The two *Fth* subclades B.71 and B.72 (within B.12), can be distinguished using the B.72 set which showed an amplification curve for all clades and subclades except of subclade B.71 (Table 2). In conclusion (see Fig. 2 and Figure S1), a *Fth* B.4 strain like Fth-41 is positive for all sets (B.6, B.12 and B.71); *Fth* B.6 strains (like A-635, A-660, A-981, A-1007, A-1158) are positive for B.6 and B.72 set; *Fth* B.72 strains (like *Fth* LVS, A-271, A-663, A-702, A-1308, A-1559) are positive for B.12 and B.71, and B.71 strains (like A-317, A-702, A-1341) are only positive for B.12 (see Fig. 2 and Figure S1).

**Table 2:** Specificity of the qPCR assays. Qualitative results of qPCR are indicated as positive ( +) or negative

*Francisellaceae* species	Strain	F-16S	B.6	B.12	B.71	*Fth* clade
*Allofrancisella sp.*	W12-1067	+	-	-	-	na
*A. frigidaquae*		+	-	-	-	na
*A. guangzhouensis*		+	-	-	-	na
*A. inopinata*		+	-	-	-	na
*Parafrancisella adeliensis*		+	-	-	-	na
*Pseudofrancisella* sp.	U-3452	+	-	-	-	na
*Pseudofrancisella* sp.	U-3454	+	-	-	-	na
*F. endociliophora*	FSC 1006	+	-	-	-	na
*F. halioticida*		+	-	-	-	na
*F. hispaniensis*	FSC 454	+	-	-	-	na
*F. hispaniensis*	3523	+	-	-	-	na
*F. noatunensis*		+	-	-	-	na
*F. noatunensis*		+	-	-	-	na
*F. novicida*	Fx1	+	-	-	+	na
*F. novicida*	U112	+	-	+	+	na
*F. opportunistica*		+	-	-	-	na
*F. orientalis*		+	-	-	-	na
*F. persica*		+	-	-	-	na
*F. philomiragia*	ATCC 25015	+	-	-	-	na
*F. philomiragia*	25,01ATCC 25016	+	-	-	-	na
*F. philomiragia*	ATCC 25017	+	-	-	-	na
*F. philomiragia*	ATCC 25018	+	-	-	-	na
*F. philomiragia*	18844	+	-	-	-	na
*Ftm*	FSC 147	+	+	+	+	na
*Ftt*	ATCC 6223	+	-	-	+	na
*Fth*	LVS	+	-	+	+	B.12/B.72
*Fth*	Fth-41	+	+	+	+	B.4
*Fth*	A-271	+	-	+	+	B.12/B.72
*Fth*	A-317	+	-	+	-	B.12/B.71
*Fth*	A-635	+	+	-	+	B.6
*Fth*	A-660	+	+	-	+	B.6
*Fth*	A-663	+	-	+	+	B.12/B.72
*Fth*	A-702	+	-	+	-	B.12/B.71
*Fth*	A-981	+	+	-	+	B.6
*Fth*	A-1007	+	+	-	+	B.6
*Fth*	A-1158	+	+	-	+	B.6
*Fth*	A-1308	+	-	+	+	B.12/B.72
*Fth*	A-1341	+	-	+	-	B.12/B.71
*Fth*	A-1559	+	-	+	+	B.12/B.72
*Fth*	A-2219	+	+	-	+	B.6
*Fth*	A-2255	+	-	+	-	B.12/B.71
*Fth*	A-2299	+	+	-	+	B.6
*Fth*	Ft-42	+	+	+	+	B.4
*Fth*	A-362–15	+	+	+	+	B.4
*Bacillus cereus*^a^		-	-	-	-	na

^a^All other bacteria listed in Table S2 were negatively tested in all qPCR assays; na: not applicable; + : positive qPCR result with Ct-value below 40; -: negative PCR result without Ct-value

This PCR assay allows a clade classification from all clinical samples such as skin ulcers, pharyngeal exudates and lymph node biopsies, independently of the isolation of a bacterial *Fth* isolate. Thus, the results of this assay could be used for further investigations and epidemiological utilization. It is known that, for example, B.6 strains are erythromycin sensitive and are found more commonly in the southeastern parts of Germany, in contrast to B.12 strains, which are more common in the northwestern parts [25, 44, 45, 50]. Furthermore, as mentioned above, it was shown that B.6 and B.12 strains have different phenotypes [42, 43, 48] and they also may differ in their virulence [25, 49–52]. In the context of surveillance and epidemiological research, the collection of as much phylogenetic data as possible from a wide range of tularemia cases is of significant importance. The assay described here offers a valuable solution, as it allows for the independence of bacterial isolates, providing notable benefits over SNP-based tools that rely mainly on whole genome sequencing of bacterial isolates. Further studies are needed to analyse the association of specific clades with distinct clinical manifestations and geographical distribution in Germany.

### Multiplex qPCR assay for diagnostic or environmental investigations

As a next step, we evaluated the primer/probe sets for diagnostic and environmental samples (ticks) in multiplex qPCR assays. So far, in our consultant laboratory the RD1 conventional PCR is used to identify the *F. tularensis* subspecies [32]. By RD1 PCR, amplicons are only available if the concentration of the target DNA is higher than 10^4^ GE (data not shown). Therefore, the qPCR Panel 1 was implemented to recognize all *Francisellaceae* representatives (using the F-16S set), and to detect specifically the subspecies *Fth*, representing the major causative agent of tularemia in Germany, by using the Ft-tul4 (*F. tularensis*-specific, [46, 62] and Fth-B2 (*Fth*-specific [34],) set (see Fig. 2). The Ft-tul4 set targets gene encoding the single copy Tul4 surface protein-encoding gene and is commonly used to detect all three *F. tularensis* subspecies as well as *F. novicida* [46, 62] The Fth-B2 set targets a hypothetical gene (locus tag FTS_0806 in *Fth* FSC200) and has been shown to specifically identify the *F. tularensis* subspecies *holarctica* with a high sensitivity (limit of detection: 5 fg, [34]). The sensitivity of all three primer/probe sets (F-16S, Ft-tul4 and Fth-B2) was found to be comparable (data not shown). In total, 22 clinical and 8 tick samples were tested and the results are given in Table 3. The qPCR panel 1 successfully and specifically detected the subspecies *Fth* in all positive samples. False positive results were not observed since all negative samples did not show an amplification signal (no threshold cycle (Ct)-value). The identification of the subspecies *Fth* was also successful when the bacterial DNA concentration was low (< 10^4^ GE, data not shown) and the RD1 PCR was unsuccessful, which was shown for sample A-2087–2. However, using the *Francisellaceae*-specific qPCR, we now would also be able to detect opportunistic *Francisellaceae* species in humans with tularemia-like symptoms. There are case reports from Europe and yet also one case from Germany, reporting tularemia-like infections in humans due to opportunistic *Francisella* species [18, 26, 27, 63]. Thus, it is important to introduce qPCR tests for the detection of such opportunistic *Francisella* species for the analysis of tularemia and “tularemia-like” patients in Germany.

**Table 3:** Clinical and tick samples used in qPCR panel 1 and panel 2. Qualitative results of qPCR are indicated as positive ( +) or negative (-)

Sample ID	Sample type	Panel 1^a^			Panel 2			
		F-16S	Ft-tul4	Fth-B2	B.6	B.12	B.71	*Fth* clade
A-1811–2	human	-	-	-	-	-	-	na
A-1888	human	+	+	+	+	-	+	B.6
A-2087–2	human	+	+	+	-	+	+	B.12/B.72
A-2119	human	+	+	+	+	-	+	B.6
A-2123–3	human	+	+	+	-	+	+	B.12/B.72
A-2131–2	human	-	-	-	na	na	na	na
A-2132–1	human	-	-	-	-	-	-	na
A-2139	human	-	-	-	-	-	-	na
A-2150–3	human	-	-	-	-	-	-	na
A-2160–2	human	+	+	+	+	-	+	B.6
A-2161	human	+	+	+	+	-	+	B.6
A-2174	human	+	+	+	+	-	+	B.6
A-2175–2	human	-	-	-	-	-	-	na
A-2182–2	human	-	-	-	na	na	na	na
A-2196	human	-	-	-	-	-	-	na
A-2220–2	human	+	+	+	+	-	+	B.6
A-2226–1	human	+	+	+	+	-	+	B.6
A-2228–2	human	+	+	+	+	-	+	B.6
A-2237–2	human	-	-	-	na	na	na	na
A-2254–2	human	-	-	-	na	na	na	na
A-2257–2	human	-	-	-	-	-	-	na
A-2288	human	-	-	-	-	-	-	na
tick-1	*D. reticulatus*	+	-	-	na	na	na	na
tick-2	*D. reticulatus*	+	-	-	na	na	na	na
tick-3	*D. reticulatus*	+	-	-	na	na	na	na
tick-4	*D. reticulatus*	+	-	-	na	na	na	na
tick-5	*I. ricinus*	-	-	-	na	na	na	na
tick-6	*I. ricinus*	-	-	-	na	na	na	na
tick-7	*I. ricinus*	-	-	-	na	na	na	na
tick-8	*I. ricinus*	-	-	-	na	na	na	na

^a^Internal amplification control (KoMa, see Table 1) was additionally used in panel 1; na = not applicable; + : positive qPCR result with Ct-value below 40; -: negative PCR result without Ct-value

In addition to the human samples, we also tested DNA samples obtained from four *Ixodes ricinus* and four *Dermacentor reticulatus* ticks using the qPCR panel 1. All *D. reticulatus* samples were tested positive for F-16S, but negative for Ft-tul4 and Fth-B2 sets indicating that *F. tularensis* subspecies (including *F. novicida*) were not present, but FLEs or other *Francisellaceae* strains could be detected within these tick samples (Table 3). The F-16S Ct-values ranged between 17 and 22 indicating a high concentration of DNA. FLEs are endosymbionts of several ticks and they are more closely related to *F. tularensis* than to other *Francisella* species, like *F. philomiragia* or *F. hispaniensis*, hence belonging to clade 1 of *Francisella* species [3, 7, 15]. Since it is known that FLEs are strongly associated with *D. reticulatus* ticks compared to *I. ricinus* [3], it could be assumed that the *D. reticulatus* ticks might harbor FLEs. However, further investigations are needed to confirm this hypothesis. Moreover, tick-transmitted tularemia cases in humans also frequently occurred in Germany [3, 64]. However, less is known about the presence of *Francisellaceae* in ticks in Germany [65–67] and thus further investigations are needed to close this gap of knowledge. Therefore, we here established a fast-diagnostic qPCR tool to detect all strains of the *Francisellaceae* genera (at least of the genera *Francisella, Parafrancisella, Allofrancisella and Pseudofrancisella*) to be able to detect opportunistic *Francisella* species in human samples in addition to *Fth* strains (see Fig. 2, panel 1). Most of the published assays were established to detect *F. tularensis* and *F. tularensis* subspecies ( [33, 34, 68]; see introduction) or to detect *Francisella* species from (complex) samples [34, 35, 39–41], for example based on Next Generation Sequencing (NGS) [39]. But they were not generated for a fast and easy detection of all *Francisellaceae* genera.

Due to molecular-epidemical interests, it is highly valuable to identify the *Fth* (sub)clade within a *Fth*-positive sample (Fig. 2, panel 2). Therefore, the qPCR panel 2 was implemented to easily discriminate the basal clades B.4, B.6 and B.12 as well as the subclades B.71 and B.72 (in clade B.12) by using one qPCR reaction (triplex qPCR) composed of the B.6, B.12 and B.72 sets. Using panel 2, we were able to identify the subclades of ten *Fth*-positively tested samples (Table 3): eight samples belonging to *Fth* B.6 clade and two samples to B.72. In addition to the clinical samples, also all strains of *Francisellaceae* DNAs listed in Table S1 were tested with the panel 1 and panel 2 showing robust and stable performance of the qPCR assays.

## Conclusion

In this study we successfully implemented two fast, easy and robust qPCR assays (see Fig. 2): (1) Panel 1 provides the detection of all known *Francisellaceae* strains which improves the PCR-based diagnostics. (2) Panel 2 can be used for epidemiological research discriminating the *Fth* clades, B.4, B.6, B.71 and B.72, which are commonly found in Germany. Panel 2 thus serves as the basis for investigating the association between specific clades and distinct clinical manifestations.

## Supplementary Information

Supplementary Material 1.

## Data Availability

All data generated or analysed during this study are included in this published article [and its supplementary information files].
