# Supplementary material for: Detection of Francisellaceae and the differentiation of main European F. tularensis ssp. holarctica strains (Clades) by new designed qPCR assays
Source: BMC Microbiol. 2025 Jan 17;25:28. doi: 10.1186/s12866-025-03751-9 (PMC11740472; doi:10.1186/s12866-025-03751-9)
Supplement: Supplementary file 1 — Supplementary Material 1. [file 12866_2025_3751_MOESM1_ESM.docx]

**Detection of *Francisellaceae* and the differentiation of main European *F. tularensis* ssp. *holarctica* strains (Clades) by new designed qPCR assays**

Kristin Köppen^1^, Kerstin Rydzewski^1^, Julia Zajac^1^, Marwah Al-Senwi^1^, Sema Evcimen^1^, Darius Schulze^1^, Daniela Jacob^2^, Klaus Heuner^1,2*^

*^1^ Working group: Cellular Interactions of Bacterial Pathogens, Centre for Biological Threats and Special Pathogens, Highly Pathogenic Microorganisms (ZBS 2), Robert Koch Institute, Berlin, Germany*

*^2^ National Consultant Laboratory for Francisella tularensis, Centre for Biological Threats and Special Pathogens, Highly Pathogenic Microorganisms (ZBS 2), Robert Koch Institute, Berlin, Germany*

*Corresponding author:

Dr. Klaus Heuner

Cellular Interactions of Bacterial Pathogens

Highly Pathogenic Microorganisms (ZBS 2)

Centre for Biological Threats and Special Pathogens

Robert Koch Institute, Seestraße 10

13353 Berlin/Germany

Phone: +49 30 18754-2226

E-mail: [HeunerK@rki.de](mailto:HeunerK@rki.de)

**to be submitted to BMC Microbiology**

**Table S1**: *Francisellaceae* strains used in this study.

| **Species** | **strain** | **reference** |
| --- | --- | --- |
| *Allofrancisella* sp. | W12-1067 | [18] |
| *A. frigidaquae* |  | DSM 101835 |
| *A. guangzhouensis* |  | DSM 102975 |
| *A. inopinata* |  | DSM 101834 |
| *F. endociliophora* | FSC1006 | Sjödin 2014 |
| *F. halioticida* |  | DSMZ 101678 |
| *F. hispaniensis* | FSC454 | DSM 22475 |
| *F. hispaniensis* | 3523 | Whipp 2003 |
| *F. noatunensis* |  | DSM 18777 |
| *F. noatuniensis* |  | DSMZ 23596 |
| *F. novicida* | Fx1 | FSC 156 |
| *F. novicida* | U112 | ATCC 15482 |
| *F. opportunistica* |  | DSMZ 107100 |
| *F. orientalis* |  | DSMZ 21254 |
| *F. persica* |  | DSMZ 23729 |
| *F. philomiragia* | 25015 | ATCC 25015 |
| *F. philomiragia* | 25016 | ATCC 25016 |
| *F. philomiragia* | 25017 | ATCC 25017 |
| *F. philomiragia* | 25018 | ATCC 25018 |
| *F. philomiragia* | 18844 | Froböse 2020 |
| *F. tularensis* ssp. *holarctica* | LVS | ATCC 29684 |
| *F. tularensis* ssp. *holarctica* | Fth-41 | [32] |
| *F. tularensis* ssp. *holarctica* | A-271 / FDC408 | [32] |
| *F. tularensis* ssp. *holarctica* | A-317 / FDC409 | [32] |
| *F. tularensis* ssp. *holarctica* | A-635 | [32] |
| *F. tularensis* ssp. *holarctica* | A-660 | [32] |
| *F. tularensis* ssp. *holarctica* | A-663 | [32] |
| *F. tularensis* ssp. *holarctica* | A-702 | [32] |
| *F. tularensis* ssp. *holarctica* | A-981 | [32] |
| *F. tularensis* ssp. *holarctica* | A-1007 | [32] |
| *F. tularensis* ssp. *holarctica* | A-1158 | [32] |
| *F. tularensis* ssp. *holarctica* | A-1308 | [32] |
| *F. tularensis* ssp. *holarctica* | A-1341 | [32] |
| *F. tularensis* ssp. *holarctica* | A-1559 | [32] |
| *F. tularensis* ssp. *holarctica* | A-2219 | ZBS 2 |
| *F. tularensis* ssp. *holarctica* | A-2255 | ZBS 2 |
| *F. tularensis* ssp. *holarctica* | A-2299 | ZBS 2 |
| *F. tularensis* ssp. *holarctica* | Ft-42 / CCUG 17316 | CCUG |
| *F. tularensis* ssp. *holarctica* | A-362-15 | IZSLER, Italy |
| *F. tularensis* ssp. *mediasiatica* | FSC147 | FOI, Sweden |
| *F. tularensis* ssp. *tularensis* | 6223 | ATCC 6223 |
| *Parafrancisella adeliensis** | FSC 1327 | FOI, Sweden |
| *Pseudofrancisella* sp. | U-3452 | LANUV NRW |
| *Pseudofrancisella* sp. | U-3454 | LANUV NRW |

* published as *Francisella* *adeliensis* [53], but now listed as *Parafrancisella adeliensis* [4].FOI: Swedish Defence Research Agency; LANUV NRW: Landesamt für Natur, Umwelt und Verbraucherschutz, Nordrhein-Westfalen, Germany; CCUG: Culture Collection University of Gothenburg, Sweden; IZSLER: Istituto Zooprofilattico Sperimentale della Lombardia e dell'Emilia Romagna, Italy

**Table S2**: Other bacterial strains used in this study. *

| **Species** |
| --- |
| *Aeromonas veronii* |
| *Bacillus cereus* |
| *Bacillus thuringiensis* |
| *Bordetella bronchiseptica* |
| *Brucella melitensis* |
| *Burkholderia mallei* |
| *Citrobacter freundii* |
| *Enterococcus faecium* |
| *Escherichia coli* |
| *Geobacillus stearothermophilus* |
| *Klebsiella oxytoca* |
| *Klebsiella pneumoniae* |
| *Legionella pneumophila* Corby |
| *Ochrobactrum anthropi* |
| *Proteus mirabilis* |
| *Pseudomonas aeruginosa* |
| *Pseudomonas fluorescens* |
| *Pseudomonas putida* |
| *Salmonella enterica* subsp*. enterica* serovar Typhimurium |
| *Serratia marcescens* |
| *Shigella flexneri* |
| *Staphylococcus aureus* |
| *Vibrio cholerae* |
| *V. parahaemolyticus* |
| *V. vulnificus* |
| *Yersinia enterocolitica* |

* DNA provided by the German National Consultant Laboratory for Tularemia in human medicine (ZBS 2, Robert Koch Institute, Berlin)

**Table S3**: Additional oligonucleotides and probes sequences, which were rejected

| Name | probe or primer | sequence (5´-> 3´) | Target |
| --- | --- | --- | --- |
| F-16S-F1 | forward primer | gggataccagttggaaacgac | 16S |
| F-16S-R1 | reverse primer | ttgggccgtgtctcagtcc |  |
| F-16S-P1 | probe | Cy5.5-gctaatccaacgcaggctcatccat-BHQ-3 |  |
| F-30S-F3 | forward primer | ggtggtagggtwaaagatttgcca | rpsL,  FTL_0232,  30S |
| F-30S-R3 | reverse primer | taagacttaggacgctttgtaccata |  |
| F-30S-P3 | probe | Cy5.5-gtgctttagatacttcaggtgttaataatcgtaagc-BHQ-3 |  |
| B6-D-F | forward primer | gccagtatcaattcagattaataaaggctta | FTL_0701 |
| B6-D-R | reverse primer | aatctctggagtcatctttggc |  |
| B6-D2-F | forward primer | caattcagattaataaaggcttagctta |  |
| B.6-D-P | probe | Cy5-attaactaaacagacatctatgcatccaggtgttt -BHQ-2 |  |
| B.12-A-F | forward primer | actggtgacatgaatgccattattatg | FTL_0742 |
| B.12-A-R | reverse primer | agtttcgcctttacgtactgttg |  |
| B.12-A2-F | forward primer | actggtgacatgaatgccattatt |  |
| B.12-A3-F | forward primer | tctaccactggtgacatgaatgc |  |
| B.12-A2-R | reverse primer | tcgcctttacgtactgttgct |  |
| B.12-A-P | probe | FAM-gtggtaagttttcgagctgacgaggattgg-BHQ-1 |  |
| B.12-B-F | forward primer | taccaaatagagattattatttagatgaaaacg | FTL_0734 |
| B.12-B-R | reverse primer | aactcttcttgcagaaatccagtt |  |
| B.12-C-F | forward primer | gcttgagtcataggtggcac | FTL_1896 |
| B.12-C-R | reverse primer | taacaagtgtaggtgcagcaac |  |
| B.71-F1 | forward primer | gattttggacttaggaatggttttgttatc | FTL_0147 |
| B.71-R1 | reverse primer | acgatatattcgtaactgatccaggag |  |
| B.71-P1a | probe | Hex-gccaacagttacaaaaaaaggtgatgtatctaaattac-BHQ-1 |  |


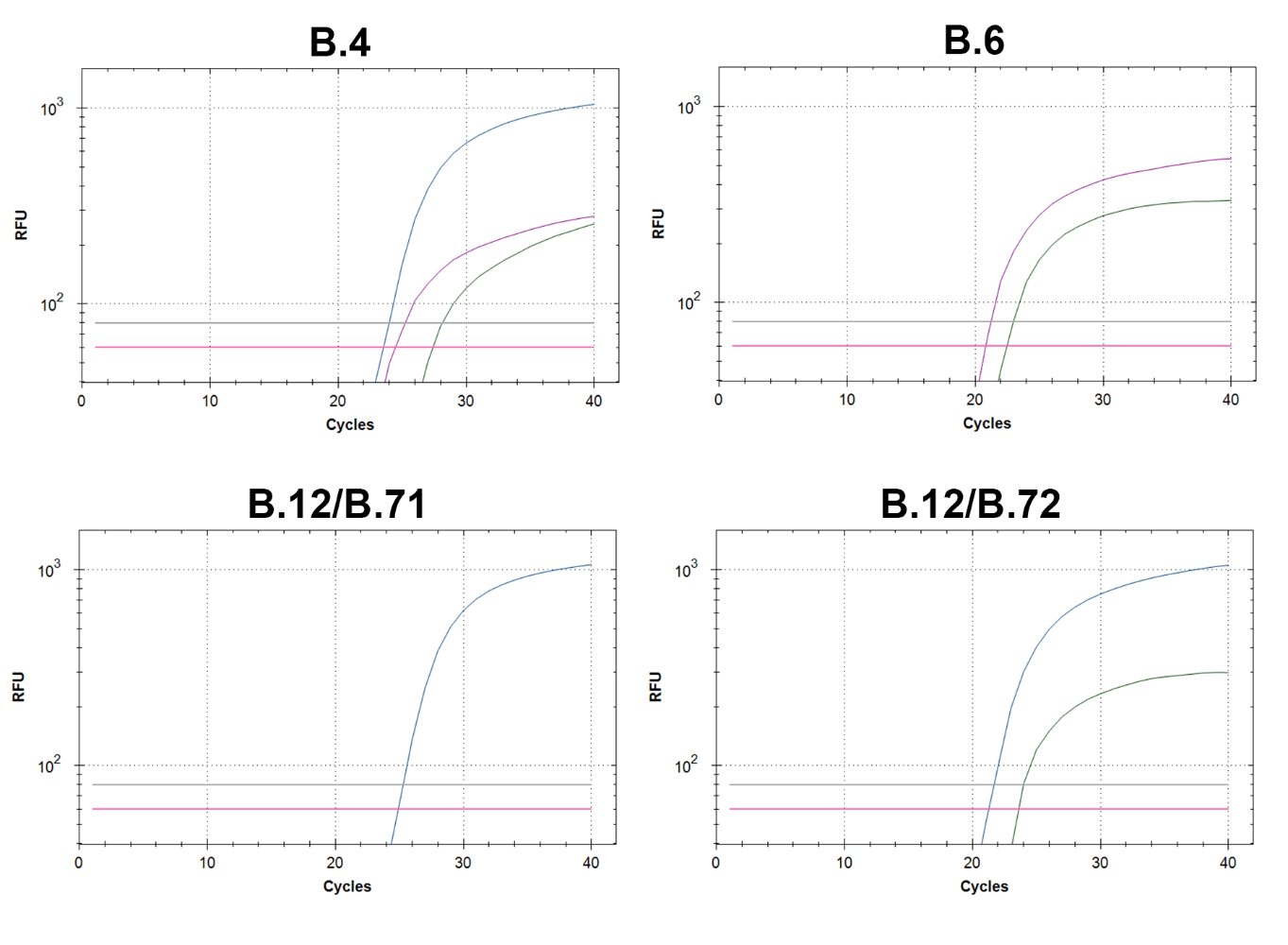


**Figure S1: *Fth* subclade discrimination using real-time PCR panel 2.** Genomic DNA of *Fth* isolates belonging to *Fth* subclade B.4 (Fth-41), B.6 (A-660), B.71 (A-2255) and B.72 (A-271) was used in real-time PCR panel 2 (see table 4 and 5). Following results were obtained: B.4 strain showed a positive signal for B.12-C-P (FAM, blue), B.6-ES-P (Cy5, purple) and B.71-P2 (Hex, green); B.6 strain was positive for B.6-ES-P (Cy5, purple) and B.71-P2 (Hex, green); B.71 strain was only positive for B.12-C-P (FAM, blue) and B.72 strain showed a positive signal for B.12-C-P (FAM, blue) and B.71-P2 (Hex, green).
